# Global diversification of a tropical plant growth form: environmental correlates and historical contingencies in climbing palms

**DOI:** 10.3389/fgene.2014.00452

**Published:** 2015-01-08

**Authors:** Thomas L. P. Couvreur, W. Daniel Kissling, Fabien L. Condamine, Jens-Christian Svenning, Nick P. Rowe, William J. Baker

**Affiliations:** ^1^Institut de Recherche pour le Développement, UMR-DIADE, MontpellierFrance; ^2^Laboratoire de Botanique Systématique et d’Ecologie, Département des Sciences Biologiques, Université de Yaoundé I – Ecole Normale Supérieure, YaoundéCameroon; ^3^Institute for Biodiversity and Ecosystem Dynamics, University of Amsterdam, AmsterdamNetherlands; ^4^Department of Biological and Environmental Sciences, University of Gothenburg, GöteborgSweden; ^5^Section for Ecoinformatics and Biodiversity, Department of Bioscience, Aarhus University, AarhusDenmark; ^6^University Montpellier 2, MontpellierFrance; ^7^CNRS, UMR AMAP, MontpellierFrance; ^8^Royal Botanic Gardens, SurreyUK

**Keywords:** ClaSSE, BAMM, growth form, lianas, plant traits, rattans, tropical rain forest evolution, Dipterocarpaceae

## Abstract

Tropical rain forests (TRF) are the most diverse terrestrial biome on Earth, but the diversification dynamics of their constituent growth forms remain largely unexplored. Climbing plants contribute significantly to species diversity and ecosystem processes in TRF. We investigate the broad-scale patterns and drivers of species richness as well as the diversification history of climbing and non-climbing palms (Arecaceae). We quantify to what extent macroecological diversity patterns are related to contemporary climate, forest canopy height, and paleoclimatic changes. We test whether diversification rates are higher for climbing than non-climbing palms and estimate the origin of the climbing habit. Climbers account for 22% of global palm species diversity, mostly concentrated in Southeast Asia. Global variation in climbing palm species richness can be partly explained by past and present-day climate and rain forest canopy height, but regional differences in residual species richness after accounting for current and past differences in environment suggest a strong role of historical contingencies in climbing palm diversification. Climbing palms show a higher net diversification rate than non-climbers. Diversification analyses of palms detected a diversification rate increase along the branches leading to the most species-rich clade of climbers. Ancestral character reconstructions revealed that the climbing habit originated between early Eocene and Miocene. These results imply that changes from non-climbing to climbing habits may have played an important role in palm diversification, resulting in the origin of one fifth of all palm species. We suggest that, in addition to current climate and paleoclimatic changes after the late Neogene, present-day diversity of climbing palms can be explained by morpho-anatomical innovations, the biogeographic history of Southeast Asia, and/or ecological opportunities due to the diversification of high-stature dipterocarps in Asian TRFs.

## INTRODUCTION

“The object of all climbing plants is to reach the light and free air with as little expenditure of organic matter as possible.”

Darwin (1865)

Besides being the most diverse terrestrial ecosystem on Earth, tropical rain forests (TRF) contain a wide array of growth forms such as large emergent to small understory trees, shrubs, epiphytes, lianas, and vines, as well as parasitic plants (Richards, 1996). Understanding how these different growth forms have originated and contributed to the diversification of TRFs through time provides important insights into the evolution of this biome (e.g., Givnish et al., 2014). Anthropogenic disturbances have caused significant structural changes in TRFs and recent studies indicate that understanding changes in the abundance and biomass of specific growth forms has important implications for future community and ecosystem dynamics in TRFs (Phillips et al., 2002; Schnitzer and Bongers, 2011).

The climbing growth form (lianas and vines) constitutes a key component of tropical forests worldwide, contributing considerably to species diversity (between 10–50%), stem density (∼25%), and ecosystem processes such as forest transpiration and carbon sequestration (Gentry, 1991; Schnitzer and Bongers, 2002). The climbing habit is present in more than 130 plant families and has evolved independently numerous times within angiosperms (Gentry, 1991). Across species from different woody plant families, abundance of climbers in tropical forests is negatively correlated with mean annual precipitation and positively with seasonality, peaking in tropical dry forests (Schnitzer, 2005). However, regional studies of climber abundance do not necessarily support such results and suggest that structural characteristics of the forests can be more important than the physical environment (van der Heijden and Phillips, 2008). There is also large variation in stem structural properties and hydraulic architectures among climbing plants (Tomlinson and Fisher, 2000; Rowe et al., 2004) that could potentially mask environmental controls of specific lineages in cross-taxon analyses.

Despite their ecological importance, few studies have investigated the role of the climbing habit in the evolution and diversification of TRF. Based on sister group comparisons of species richness between climbing and non-climbing clades within 48 angiosperm families, Gianoli (2004) inferred that the climbing habit is a key innovation within flowering plants, leading to higher species richness than non-climbing sister groups. Wang et al. (2012) used a generic level dated phylogeny of the largely climbing TRF family Menispermaceae and found evidence for a burst of diversification shortly after the Cretaceous–Paleogene boundary, suggesting an important role of the climbing habit in the diversification of TRFs throughout the Cenozoic (this is also found in ferns; Schneider et al., 2004). Moreover, biogeographic analyses of the Neotropical tribe Bignonieae (Bignoniaceae) further showed that drivers of climber diversification are possibly related to climate drying and Andean orogeny (Lohmann et al., 2013).

With about 2,500 species, palms (Arecaceae) are a species-rich, monocotyledonous plant family characteristic of tropical and subtropical ecosystems (Dransfield et al., 2008; Couvreur and Baker, 2013). Palms have limited ability to survive in areas with cold and arid climates due to structural constraints (Tomlinson, 2006). As a consequence, palm species richness generally peaks in warm and humid areas with low seasonality (Bjorholm et al., 2005; Kissling et al., 2012a). However, historical legacies related to evolutionary history of specific lineages (Baker and Couvreur, 2013a,b), dispersal limitation (Bjorholm et al., 2006; Kissling et al., 2012a; Eiserhardt et al., 2013), and the unique history of biogeographic regions (Bjorholm et al., 2006; Kissling et al., 2012a; Blach-Overgaard et al., 2013; Rakotoarinivo et al., 2013) also play an important role in shaping global patterns of palm species richness and distribution.

A large diversity of growth forms has evolved within palms, including tree palms, palms with clustered stems, acaulescent palms, and climbing palms (Dransfield, 1978; Dransfield et al., 2008; Balslev et al., 2011). The climbing habit has evolved independently several times (Baker et al., 2000a), most notably within subfamily Calamoideae (**Table 1**), but also in relatively small sets of species within subfamily Arecoideae in the Neotropical genera *Desmoncus* and *Chamaedorea*, and in the Madagascan genus *Dypsis* (**Table 1**). The mainly Southeast Asian *Calamus* is the most species-rich genus of palms and is one of the most diverse genera of climbing plants (Gentry, 1991). Climbing in palms is typically facilitated by traits such as elongated stems that are stiffened by cylindrical leaf sheaths distally, the presence of spines on almost all organs, and wider vessels compared to their self-supporting counterparts (Tomlinson and Fisher, 2000). In addition, climbing palms have evolved two unique climbing organs: the cirrus, an extension of the leaf rachis usually equipped with recurved grapnel-like spines as well and, in some taxa, hook-like reflexed leaflets, and the flagellum, a modified, sterile inflorescence also armed with recurved grapnel-like spines, which is only found in *Calamus*. Both are highly efficient attachment structures for climbing (Dransfield et al., 2008; Isnard and Rowe, 2008). However, palms do not actively twine to gain support, but rather become anchored passively on adjacent vegetation via these climbing organs (Putz, 1990).

**Table 1:** Species diversity of climbers within palm genera.

Genus (# spp./total #)	% climber	Subfamily	Regional distribution
*Calamus* (348/381)	91	Calamoideae	Afrotropics, Indomalaya, Australasia, Oceania
*Ceratolobus* (6/6)	100	Calamoideae	Indomalaya
*Chamaedorea* (1/107)	0.01	Arecoideae	Neotropics
*Daemonorops* (92/102)	90	Calamoideae	Indomalaya, Australasia
*Desmoncus* (12/12)	100	Arecoideae	Neotropics
*Dypsis* (2/144)	0.01	Arecoideae	Afrotropics (Madagascar)
*Eremospatha* (11/11)	100	Calamoideae	Afrotropics
*Korthalsia* (27/27)	100	Calamoideae	Indomalaya, Australasia
*Laccosperma* (6/6)	100	Calamoideae	Afrotropics
*Myrialepis* (1/1)	100	Calamoideae	Indomalaya
*Oncocalamus* (5/5)	100	Calamoideae	Afrotropics
*Plectocomia* (16/16)	100	Calamoideae	Indomalaya
*Plectocomiopsis* (5/5)	100	Calamoideae	Indomalaya
*Pogonotium* (2/3)	67	Calamoideae	Indomalaya
*Retispatha* (1/1)	100	Calamoideae	Indomalaya
**TOTAL (535/2445)**	**22**		

*Data are based on the World Checklist of Palms (Govaerts et al., 2014). Data for the genus *Dypsis* have been additionally updated (now two climbing species).*

As in most other monocotyledons, palms lack a vascular cambium for secondary growth. Instead, they retain their primary anatomical architecture of the stem for their entire life. Nevertheless, they can still achieve remarkable heights of up to 60 m tall (Sanin and Galeano, 2011). While some tall tree palms having developed mechanical properties to minimize elastic buckling when achieving large heights (Rich, 1986), there seems to be a critical threshold that limits palm height growth (Niklas, 1993, 1994). Thus, the tree growth form in palms could be a competitive disadvantage in tall TRF environments where structural and functional limitations prohibit them from reaching the canopy. In contrast, climbing palm stems can attain astonishing lengths, reaching up to 170 m length or even more in *Calamus*, which represents the longest unrooted aerial plant stem on record (Burkill, 1966). Given the potential importance of forest structure for abundance and distribution of climbers (van der Heijden and Phillips, 2008), it can be hypothesized that on an evolutionary timescale the global biogeographic differences in tropical rain forest canopy heights (Lefsky, 2010) might have influenced the diversification of climbing palms.

Here, we combine macroecological and macroevolutionary analyses to investigate the role of climbing in the evolutionary history of palms. We quantify global patterns and drivers of climbing palm diversity and ask what role the climbing habit has played in the diversification of palms through time. Specifically, we test the following hypotheses and corresponding predictions:

(1) The diversity of climbing palms correlates with aseasonal tropical climates and peaks in high-stature forests:
      (a) Due to physiological and functional adaptations of palms, species richness of climbing palms is positively correlated with temperature and precipitation and negatively with temperature seasonality.
      (b) Differences in canopy height among TRFs explain global variation in species richness of climbing palms, with tall forests having more climbing species than short-stature forests.
(2) The climbing habit has played an important role in palm diversification:
      (a) Diversification rates are higher for climbing than non-climbing palms lineages.
      (b) The origin of the climbing habit correlates with increases in diversification rates.

## MATERIALS AND METHODS

### PALM SPECIES DISTRIBUTION DATA

We used data on global palm species distributions from an exhaustive, authoritative checklist of the World’s palm species (Govaerts et al., 2014, data used here accessed March 2009). Though coarse in resolution, this dataset currently represents the most complete and reliable source on species distributions of all palms worldwide. The dataset records palm species presences and absences across the world within the level 3 geographic units as defined by the International Working Group on Taxonomic Databases (TDWG; Brummitt, 2001). These TDWG level 3 units mostly correspond to countries and/or major islands, such as Borneo, Madagascar, and New Guinea, but very large countries such as USA, Brazil, and China are subdivided into smaller units. We included only native palm occurrences, excluding introduced occurrences, and doubtful as well as erroneous records. To derive estimates of species richness, we summed all presences of palm species within each TDWG level 3 unit, and did this separately for climbing and non-climbing palms (see below).

The dataset contained a total of 2,445 accepted palm species names and 5,027 native occurrence records within 194 TDWG level 3 units (Kissling et al., 2012a). It does not include a recently described climbing species of *Dypsis* (Rakotoarinivo and Dransfield, 2010) which would raise the total number of liana species in this genus to two. The addition of this extra species would not impact the results presented here, but this new information was taken into account throughout the discussion and in **Table 1**.

### CATEGORIZATION OF GROWTH FORMS

We classified all palm species into two growth forms: (1) climbers, and (2) non-climbers (including all other growth forms such as stemmed and acaulescent palms). Palm species that show leaning growth forms such as some *Bactris* species (not really climbers or trees) were considered as non-climbers. For climbers, we also included palm species that can, within the same species, show both climbing and non-climbing habits (only 16 species). The remaining species (*n* = 2431) were exclusively climbers or non-climbers. Information on climbing habit was derived from the literature (Russell, 1968; Dransfield, 1979, 1986; Dransfield and Beentje, 1995; Henderson et al., 1995; Henderson, 2002, 2009; Dransfield et al., 2008; Dowe, 2010) and for a few species supplemented with expert knowledge.

### ENVIRONMENTAL DETERMINANTS OF CLIMBER AND NON-CLIMBER SPECIES RICHNESS

We tested 14 predictor variables as potential determinants of species richness in climbing and non-climbing palms. These variables reflected contemporary climate (six variables), paleoclimate (six variables), canopy height (one variable), and biogeographic region (one variable). Present and past climates (Kissling et al., 2012a; Blach-Overgaard et al., 2013; Rakotoarinivo et al., 2013) as well as biogeographic history (Baker and Couvreur, 2013a,b) are important drivers of broad-scale species distributions and diversity patterns in palms. Extracted at a coarse resolution (averaged within TDWG level 3 units), these predictor variables allow assessing how broad-scale trends in environmental conditions are related to geographic differences in species numbers of palms worldwide. We acknowledge that fine-scale heterogeneity (e.g. in climates, canopy heights etc.) within TDWG level units might not be well captured by such coarse-grained data, but analyses with new species distribution datasets at high resolution could incorporate some of this heterogeneity in the future.

#### Contemporary climate

To represent contemporary climate, we chose six climatic predictor variables from the Worldclim dataset (version 1.4; www.worldclim.org), a set of global climate layers with a spatial resolution of ca. 1 km^2^ (Hijmans et al., 2005). We used (1) annual precipitation (PREC, in mm year^-1^), (2) annual mean temperature (TEMP, in ∘C × 10), (3) precipitation seasonality measured as variation of monthly values (PREC SEAS, in mm), (4) temperature seasonality measured as standard deviation of monthly means (TEMP SEAS, in ∘C × 10), (5) extremes of drought measured as precipitation of the driest quarter (PREC DRY, in mm), and (6) extremes of cold measured as mean temperature of the coldest quarter (TEMP COLD, in ∘C × 10). Data extraction and geoprocessing of climate data are described in more detail in Kissling et al. (2012a). Several of these variables, including PREC and various temperature measures (TEMP, TEMP SEAS, TEMP COLD), have been identified as important drivers of the global range and species richness of palms (Kissling et al., 2012a). Other climate variables (e.g., minimum or maximum monthly precipitation and temperature) are highly correlated with these climatic predictors and were hence not included here.

#### Paleoclimate

To represent paleoclimatic changes over the Neogene and Quaternary epochs, we compiled both temperature and precipitation data from paleoclimatic reconstructions representing the Last Glacial Maximum (LGM, ca. 21,000 years ago), the late Pliocene (∼3 mya), and the late Miocene (∼10 mya). Data for the LGM were compiled from two climate simulations representing the Community Climate System Model version 3 (CCSM3) and the Model for Interdisciplinary Research on Climate version 3.2 (MIROC3.2), both of which were part of the second phase of the Paleoclimate Modeling Intercomparison Project^1^ (PMIP2; Braconnot et al., 2007). Paleoclimate data for deeper time periods were derived from coupled ocean–atmosphere general circulation models representing the late Pliocene (3.29–2.97 mya; Haywood et al., 2009) and the late Miocene (11.61–7.25 mya; Pound et al., 2011). All paleoclimate data were resampled in ArcGIS with a bilinear interpolation from the original resolution (2.5^′′^, 1∘, or 2.5∘) to the resolution of the contemporary climate data (see above). We then calculated anomalies (i.e., the difference between the current climate and the past) for all three time periods as well as for both precipitation and temperature data, resulting in six paleoclimatic predictor variables reflecting the change in climate since the LGM (LGM_PREC_, LGM_TEMP_), the Pliocene (PLIO_PREC_, PLIO_TEMP_), and the Miocene (MIO_PREC_, MIO_TEMP_). Anomalies were measured by subtracting the paleoclimate value in each TDWG level 3 unit from its present-day climate (i.e., contemporary climate minus paleoclimate). Large positive anomaly values indicate a higher precipitation and temperature in the present than in the past whereas small or negative anomaly values indicate the opposite, i.e., higher precipitation and temperature in the past than in the present. Hence, a negative relationship between species richness and climate anomalies indicates that species richness is higher in areas that were relatively wetter or warmer in the past than today. TDWG level 3 unit values for LGM precipitation and temperature were calculated as mean values across two paleoclimatic simulations (CCSM3, MIROC3.2). Note that temperature anomalies since the LGM to the present (LGM_TEMP_) can be considered roughly representative for climatic oscillations of the whole Quaternary (the last 2.6 million years; Jansson, 2003; Kissling et al., 2012a).

#### Canopy height

We included canopy height (CANOPY) of tropical rain forests as a predictor variable to test whether species richness of climbing palms increases with the tallness of forests. Forest canopy height data were derived from a recent global map of forest heights (Lefsky, 2010), derived from LiDAR and multispectral remote sensing data for forest patches across the world (average patch size of approximately 25 ± 50 km^2^). The product provides the 90th percentile canopy height as an index that captures the tallest heights in a given stand (Lefsky, 2010). The original dataset with a 500 m resolution was geoprocessed in ArcGIS 10 and mean values of canopy height were calculated at the resolution of the TDWG level 3 units for which the palm distribution data are available. Note that the canopy height data only represent natural forests with >70% cover and therefore leave out savannas and woodlands (Lefsky, 2010). This is unproblematic for palms which predominantly occur in tropical and subtropical forests.

#### Biogeographic history

We derived a biogeographic variable (REGION) to capture potential effects related to the long-term history of biogeographic regions. This categorical variable distinguished seven major regions: Afrotropics, Australasia, Indomalaya, Nearctic, Neotropics, Oceania, and Palaearctic (Kissling et al., 2012a). It can capture major differences in species richness and clade distributions between regions (Kissling et al., 2012a,b; Baker and Couvreur, 2013a,b) and permits examination of how species richness varies among realms once present-day environment and paleoclimate have been statistically accounted for (Kissling et al., 2012a).

#### Statistical analysis of determinants of species richness

We assessed the environmental determinants of species richness of both climbers and non-climbers with separate multi-predictor regression models. We only included TDWG level 3 units where species richness >0 and for which environmental data were available. A number of smaller islands had to be excluded for non-climbing palms because canopy height data were not available for those. Hence, final sample sizes for the statistical analysis were 82 and 164 TDWG level 3 units for climbers and non-climbers, respectively.

We used generalized linear models (GLM) with a Gaussian error distribution and included all 14 predictor variables as well as either species richness of climbers or non-climbers as response variable. We then applied a model selection based on the Akaike Information Criterion (AIC) to derive a minimum adequate model that had the smallest possible number of predictor variables (i.e., the lowest AIC value; Burnham and Anderson, 2002). We used the variance inflation factors (VIF) to test for multicollinearity among the predictors included in the regression models, excluding variables with VIF >10 before AIC model selection (TEMP SEAS and TEMP COLD for climber richness, TEMP COLD and PREC DRY for non-climber richness). All continuous predictor variables were scaled before the analysis (standardized to mean = 0 and SD = 1). We compared the relative importance of predictor variables to explain species richness of climbing and non-climbing palms with semi-standardized coefficients (for continuous variables only). Both response variables as well as several predictor variables (TEMP, PREC SEAS, TEMP SEAS, PREC DRY, LGM_TEMP_) were log-transformed to improve normality. We included second order polynomials to account for non-linear relationships if single predictor models with polynomials showed statistically significant improvements over models without polynomials (using an ANOVA model comparison at *P* < 0.05; Crawley, 2007). The reference level of the categorical variable REGION was set to the Indomalayan region because this allowed us compare all other regions to the region with the highest species richness of climbing palms (i.e. Indomalaya). For climbing palms, TDWG level 3 sample sizes for the Nearctic, Oceania, and the Palaearctic were small (*n* ≤ 1), and statistical analyses of climbing palm species richness were therefore restricted to the Afrotropics, Australasia, Indomalaya, and the Neotropics.

To test for a potential influence of spatial autocorrelation (Kissling and Carl, 2008), we further calculated Moran’s *I* values on the residuals of our minimum adequate GLMs. The geographic distance for calculating Moran’s *I* values was based on the closest neighbor of each TDWG level 3 unit, and significance of Moran’s *I* was determined by permutation tests (*n* = 10000 permutations). Moran’s *I* values of the residuals of both minimum adequate GLMs were not statistically significant (see Results) and residual spatial autocorrelation was therefore considered to be unimportant in our analyses. Hence, there was no need to additionally implement spatial regression models (Kissling and Carl, 2008). All statistical analyses on determinants of species richness were done with the R version 3.0.2 (R Development Core Team, 2013). Moran’s *I* analyses were conducted using the R library ‘spdep.’

### DIVERSIFICATION ANALYSES

Beyond examining the determinants of species richness of climbers and non-climbers, we performed diversification analyses to test whether the evolution of climbers has an impact on diversification rates as a whole and for specific clades.

#### Overall diversification analysis of climbing palms

We used the Cladogenetic State Speciation and Extinction model (ClaSSE, Goldberg and Igić, 2012) to test if climbing species have overall higher diversification rates than non-climbing palms. We generated a species-level phylogeny of palms (i.e., a 2,445 tip dated phylogeny), based on the genus-level, fossil-calibrated tree of Couvreur et al. (2011a) derived from the global palm supertree of Baker et al. (2009) as an initial backbone. This phylogenetic tree provides the most robust hypothesis of relationships between palm genera to date. We then simulated random species phylogenies at the tips (i.e., the genera) of this backbone tree using known species diversity for each genus (see above). This was done 100 times to get 100 species level trees. We used the script “gratfMissingTaxa” written by François Michonneau^2^ and modified it to simulate phylogenies under a pure birth model (sim.bdtree function in APE, Paradis et al., 2004). For each genus, the crown node of the simulated species-level phylogeny was inserted either (i) randomly along the whole length of the subtending branch of the genus (referred to as the ‘non-constrained analysis’), or (ii) following a uniform distribution between 0.2 and 0.8 (i.e., between 20 and 80% of the whole length of the subtending branch, referred to as ‘constrained analysis’). This latter approach was used to test the effect of extremely young or old inferred crown node ages on the results.

Different proportions of climbers and non-climbers within genera (see **Table 1**) can create a problem when simulating random species relationships at the tips of the phylogeny because species-level relationships are unknown. A preliminary analysis suggested that coding all species within climbing genera as climbers (generic level coding) or coding the observed proportions within each genus (i.e., coding each species as is) had little effect on the results. We therefore used the generic level coding approach for subsequent analyses.

**FIGURE 1:**
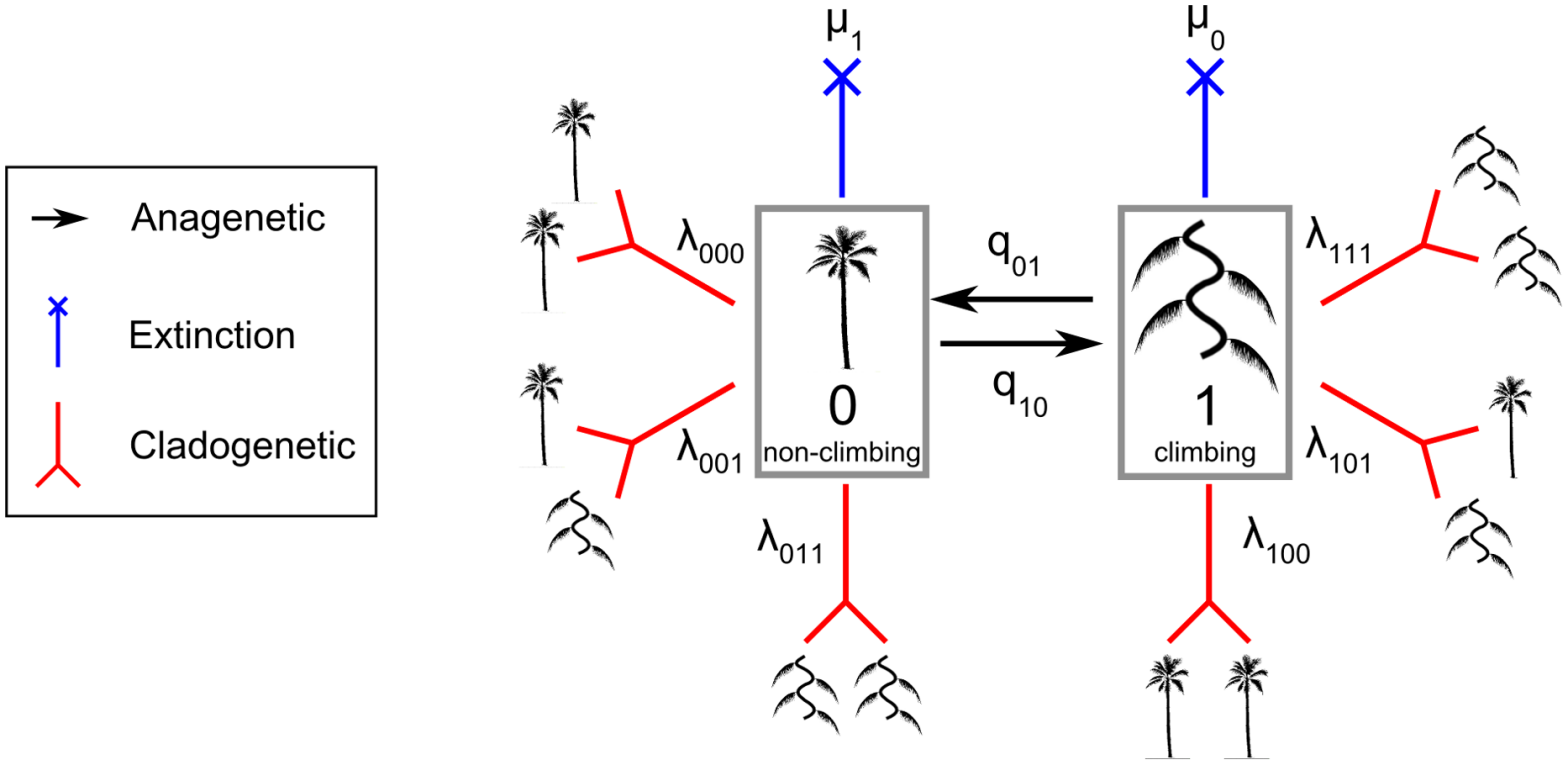
**The Cladogenetic State Speciation and Extinction model (ClaSSE) as implemented in this study for climbers and non-climbers in palms.** The figure highlights the 10 model parameters: two anagenetic changes (q_01_ and q_10_ along the branches), six cladogenetic changes (λ_000_, λ_111_, λ_110_, λ_001_, λ_100_, λ_011_; at the node) and two extinctions (μ_1_ and μ_2_). See text for details.

The 100 trees generated above were combined with the trait dataset of growth forms. The ClaSSE model is a derived model of the binary state speciation and extinction model (BiSSE) (Maddison et al., 2007). The ClaSSE model has ten parameters (**Figure 1**): two cladogenetic speciation rates without character change associated with the non-climbing habit (0) [λ_000_; the abbreviation means that one lineage with state 0 gives two lineages with state 0 and 0 (000)] and the climbing habit (1) (λ_111_), four cladogenetic speciation rates with character change: (λ_110_), (λ_001_), (λ_100_), and (λ_011_), two extinction rates associated with non-climbing (μ_1_) and climbing (μ_0_), and two anagenetic state change rates with one from climbing to non-climbing (q_10_) and one from non-climbing to climbing (q_01_). For clarity, we call the speciation rate that produces two daughter lineages from one parent without character changes (λ_000_ and λ_111_) the “symmetrical” speciation rate, and the speciation rate that produces two daughter lineages from one parent with character changes (λ_110_, λ_001_, λ_100_, and λ_011_) the “asymmetrical” speciation rate. Overall, speciation rates for a given growth form are the sum of symmetrical and asymmetrical speciation rates estimated by the ClaSSE models.

To identify the best model given our data we considered ten ClaSSE diversification scenarios (**Figure 1**):

(1) Null model (three free parameters): diversification rates are independent of growth form (λ_111_ = λ_000_ = λ_110_ = λ_001_ = λ_100_ = λ_011_, μ_1_ = μ_0_, and q_10_= q_01_),
(2) Cladogenetic speciation model (eight free parameters): growth form only affects cladogenetic speciation (λ_111_ ≠ λ_000_ ≠ λ_110_ ≠ λ_001_ ≠ λ_100_ ≠ λ_011_, μ_1_= μ_0_, and q_10_= q_01_),
(3) Symmetrical cladogenetic speciation model (five free parameters): growth form only affects symmetrical cladogenetic speciation (λ_111_ ≠ λ_000_, λ_110_ = λ_001_ = λ_100_ = λ_011_, μ_1_= μ_0_, and q_10_= q_01_),
(4) Asymmetrical cladogenetic speciation model (seven free parameters): growth form only affects asymmetrical cladogenetic speciation (λ_111_ = λ_000_, λ_110_ ≠ λ_001_ ≠ λ_100_ ≠ λ_011_, μ_1_= μ_0_, and q_10_= q_01_),
(5) Anagenetic state change model (four free parameters): growth form only affects anagenetic state change (λ_111_ = λ_000_ = λ_110_ = λ_001_ = λ_100_ = λ_011_, μ_1_ = μ_0_, and q_10_ ≠ q_01_),
(6) Extinction model (four free parameters): growth form only affects the extinction rate (λ_111_ = λ_000_ = λ_110_ = λ_001_ = λ_100_ =λ_011_, μ_1_ ≠ μ_0_, and q_10_= q_01_),
(7) Cladogenetic speciation and extinction model (nine free parameters): growth form affects cladogenetic speciation and extinction (λ_111_ ≠ λ_000_ ≠ λ_110_ ≠ λ_001_ ≠ λ_100_ ≠ λ_011_, μ_1_ ≠ μ_0_, and q_10_= q_01_),
(8) Anagenetic state change and extinction model (five free parameters): growth form affects anagenetic state change and extinction (λ_111_ = λ_000_ = λ_110_ = λ_001_ = λ_100_ = λ_011_, μ_1_ ≠ μ_0_, and q_10_ ≠ q_01_),
(9) Cladogenetic and anagenetic state change model (nine free parameters): growth form affects both cladogenetic speciation and anagenetic state change (λ_111_ ≠ λ_000_ ≠ λ_110_ ≠ λ_001_ ≠ λ_100_ ≠ λ_011_, μ_1_= μ_0_, and q_10_ ≠ q_01_),
(10) Full model (10 free parameters): components of the diversification rate are dependent on the growth form (λ_111_ ≠ λ_000_ ≠ λ_110_ ≠ λ_001_ ≠ λ_100_ ≠ λ_011_, μ_1_ ≠ μ_0_, and q_10_ ≠ q_01_).

All analyses on both datasets were performed using the R-package *diversitree* 0.7–6 (FitzJohn, 2012). For each tree, we computed the AIC_c_ (AIC corrected for finite sample sizes) corresponding to each ClaSSE model. In addition, we evaluated the support for the selected model against all models nested within it using the likelihood ratio test (LRT, significant at *P* < 0.05). The scenario supported by the LRT and with the lowest AIC_c_ value was considered the best given the data over all 100 trees. We did not undertake Markov Chain Monte Carlo (MCMC) analyses to estimate the confidence intervals of the parameters because these are highly dependent on each individual tree shape and thus have no real meaning in our context (randomly generated species level trees). In contrast, the maximum likelihood (ML) analyses are averaged over all trees and here provide a better way to take the phylogenetic uncertainty into account.

#### Clade specific diversification analyses

In order to test for possible shifts in diversification rates (speciation minus extinction) associated with the evolution of the climbing habit we used the Bayesian Analysis of Macroevolutionary Mixtures approach implemented in BAMM version 2.2.0 (Rabosky, 2014). This analysis is complementary to ClaSSE in that BAMM tests for rate shifts across lineages whereas ClaSSE investigates the significance of the climbing habit for the diversification of the family as a whole.

The main assumption of previous methods for identifying diversification rate shifts in phylogenies was based on constant rates between the shifts (e.g., MEDUSA, Alfaro et al., 2009). This assumption is generally violated, especially in large trees (Morlon et al., 2011; Morlon, 2014). BAMM explicitly accounts for rate variation through time and uses a reversible jump MCMC algorithm to quickly explore numerous candidate models of lineage diversification (Rabosky, 2014). BAMM has been shown to better identify increases/decreases in diversification shifts when compared to other methods such as MEDUSA (Rabosky, 2014).

BAMM accommodates incomplete taxon sampling under a phylogenetically structured sampling. In our phylogeny, we have included most palm genera, each one being represented by a single species. We thus provided BAMM with the proportion of species sampled per genus (i.e., 1/number of species in genus). We used the chronogram of palm genera as the input tree (Couvreur et al., 2011a). Priors were estimated with BAMMTools (Rabosky et al., 2014b) using the function “setBAMMpriors”. A compound Poisson process is implemented in BAMM for the prior probability of a rate shift along any branch. A prior value of 1.0 suggests a strong assumption of no rate shifts across the phylogeny. However, prior studies using MEDUSA strongly suggest rate heterogeneity in palms (Baker and Couvreur, 2013b). We therefore ran our analyses under two different priors: (1) a value of 1.0, mainly to re-test the hypotheses that palms underwent a rate shift at least once in their evolutionary history, and (2) a value of 0.1 (which generates a more flattened distribution around six rate shifts) reflecting our prior knowledge and from which we derived our conclusions. In each case, we ran three independent MCMC for 1.5 million generations sampling event data every 1000 steps. After checking for convergence of parameter estimates using the effective sampling size (ESS), we re-ran the analysis for 5 million generations, sampling every 5000 steps.

Post run analyses were undertaken in BAMMTools following Rabosky et al. (2014a). To visualize where in the tree the shifts occurred, we generated the mean phylorate plot which represents the mean diversification rate (option spex = “se” in plot.bammdata) sampled from the posterior at any point in time along any branch of the phylogenetic tree (Rabosky et al., 2014a).

In contrast to methods that identify a single best rate shift configuration across a tree (e.g., MEDUSA), BAMM identifies a set of most credible rate shift sets (CSS) ordering them by posterior probability (Rabosky et al., 2014a). Here, we selected the CSS based on a Bayes factor (BF) of 50 or more. Even though the BAMM website^3^ suggests that a BF of five provides substantial evidence against the null hypothesis (no rate shift along a branch), we follow the widely cited table of Kass and Raftery (1995) where strong support should be concluded from BF between 20 and 150.

Finally, we scaled the phylogenetic tree to be proportional to the BF and marginal probabilities of a rate shift along a branch. This helps to visualizes the topological location of diversification rate shifts.

### ANCESTRAL CHARACTER RECONSTRUCTION

To identify how many times and when the climbing habit arose in palms above the genus level, we conducted an ancestral character reconstruction using a stochastic character (posterior) mapping approach, as implemented in the program SIMMAP (Huelsenbeck et al., 2003; Bollback, 2006). We used the generic level chronogram of Couvreur et al. (2011a; 183 tips) and the genus-level coded dataset (derived from the monomorphic dataset used for the diversification analyses above). This approach does not take into account the evolution of climbers within mainly non-climbing genera such as *Dypsis* and *Chamaedorea* (three species only). We also coded the genera *Calamus* and *Daemonorops* (subfamily Calamoideae; subtribe Calaminae) as ancestrally climbing, even though a few species within each genus are non-climbers (**Table 1**). Ancestral states were estimated using the *make.simmap* function in the R package phytools (Revell, 2012). Because specifying incorrect prior values can influence posterior mapping results (Couvreur et al., 2010) we used the empirical approach function of *make.simmap* where the priors (α and β) of the transformation rate from one character to another (γ) were specified as follows: β = 5 and α = β × ML(Q), with Q being the transition matrix between both states. This was achieved using the *use.empirical = TRUE* option in *make.simmap*. We undertook 10,000 generations sampling every 100 steps. We used the function ‘DensityMap’ in Phytools to depict the changes in posterior probabilities (PP) along branches of the phylogeny.

## RESULTS

### GLOBAL SPECIES RICHNESS OF CLIMBING AND NON-CLIMBING PALMS

**FIGURE 2:**
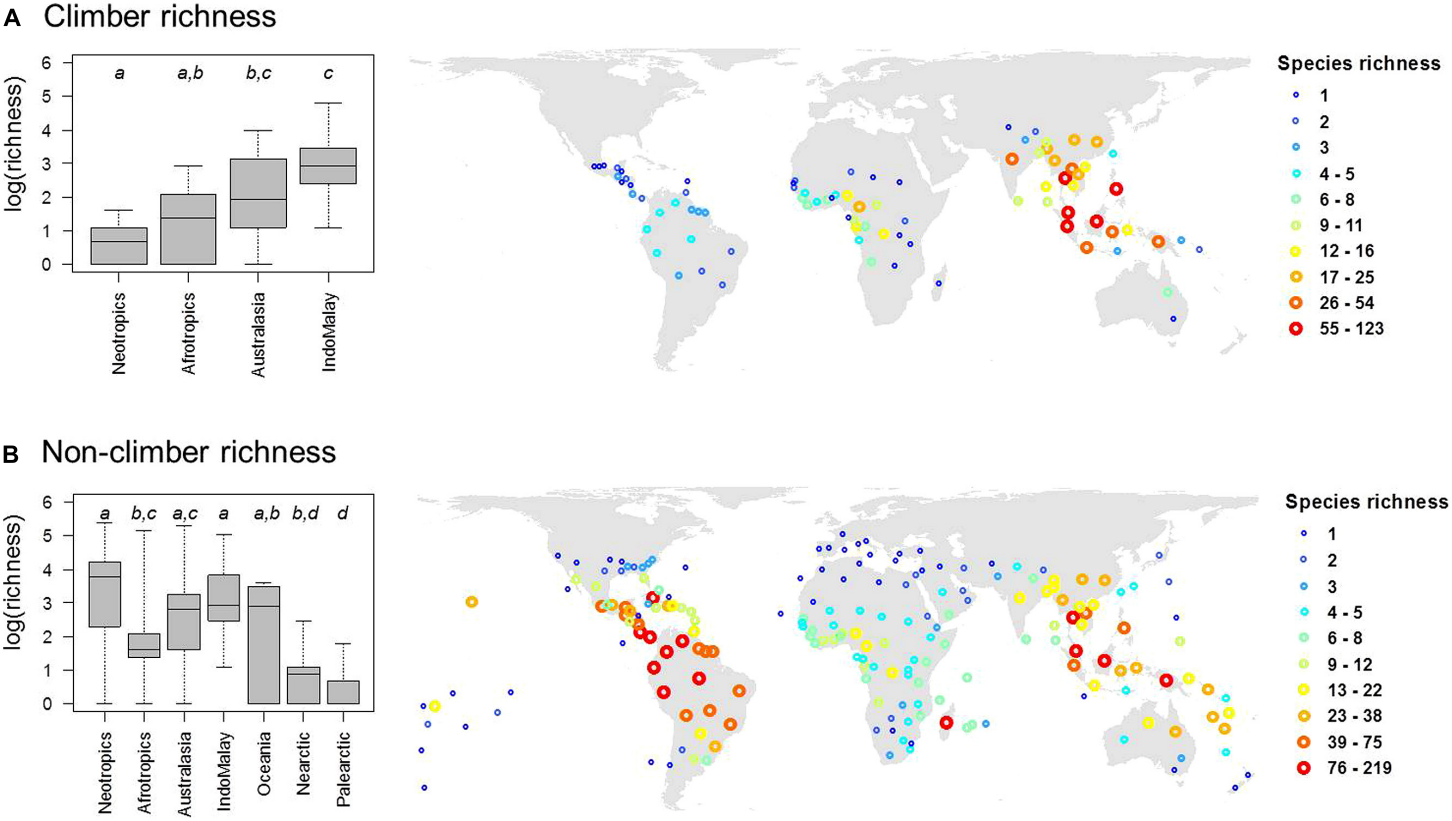
**The global distribution of species richness in **(A)** climbing and **(B)** non-climbing palms.** Global maps (in Behrmann projection) show palm species richness for the mass centroids of geographic units as defined by the International Working Group on Taxonomic Databases (TDWG level 3 units). Differences in species richness between major regions are illustrated in boxplots (left). Different letters in boxplots indicate significant differences (at *P* < 0.05) between realms as indicated by multiple pair-wise comparisons using ANOVA with a subsequent Tukey’s honestly significant difference *post hoc* test. Boxes represent the interquartile range, horizontal lines within the boxes represent medians, and whiskers extend to the most extreme data points.

Out of 2,446 palm species in our dataset, a total of 535 species (22%) were classified as climbers. The majority of climbing palm species are found within the genera *Calamus* (348 species) and *Daemonorops* (92 species). In most cases, genera with climbing habits have a high (≥90%) proportion of climbing species (**Table 1**). Geographically, climbing palm species occur in all four major tropical regions (Afrotropics, Australasia, Indomalaya, Neotropics; **Table 1**; **Figure 2**) and two species in *Calamus* even reach Oceania. Climber species richness peaked in Indomalaya, closely followed by Australasia, with the Neotropics showing the lowest climber richness (**Figure 2A**). This contrasted to the species richness of non-climbing palms, which, when compared to other tropical regions, was highest in the Neotropics and lowest in the Afrotropics (**Figure 2B**).

### DETERMINANTS OF SPECIES RICHNESS IN CLIMBING PALMS

**FIGURE 3:**
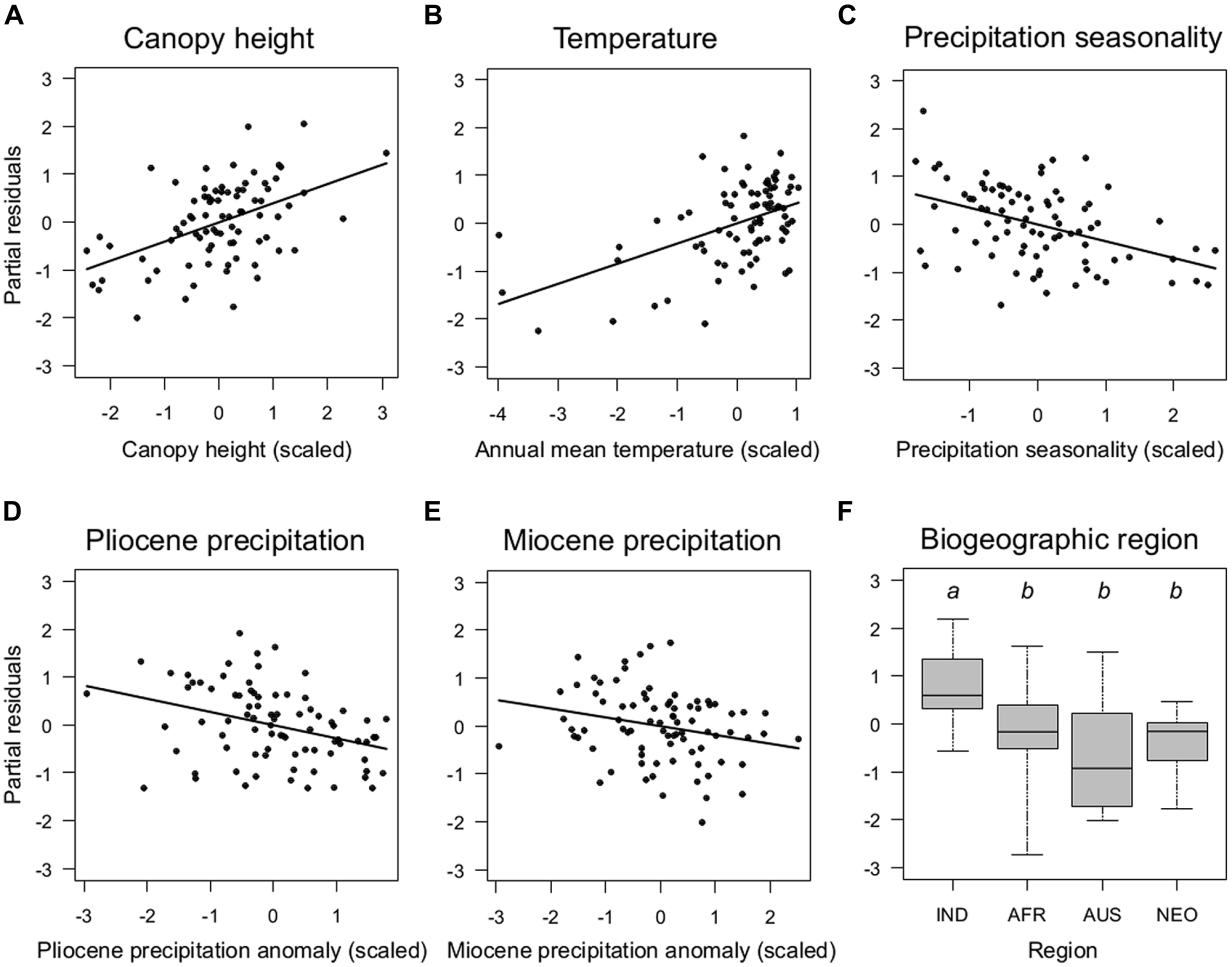
**Key determinants of the global distribution of species richness of climbing palms.** Partial residual plots show the effects of continuous **(A–E)** and categorical **(F)** predictor variables from the minimum adequate model **(Table 2)** on climbing palm species richness once all other variables in the model are statistically controlled for. Sampling units as in **Figure 2**. Details of boxplots as in **Figure 2**. Region: IND, Indomalaya; AFR, Afrotropics; AUS, Australasia; NEO, Neotropics.

Among contemporary climatic variables, TEMP (positive effect) and PREC SEAS (negative effect) were the most important variables to explain climber species richness in the minimum adequate models (**Figures 3B,C**; **Table 2**). Interestingly, PREC SEAS showed contrasting effects for climbers vs. non-climbers (negative vs. positive sign). Two additional contemporary climatic variables (PREC, TEMP SEAS) were of further importance to explain species richness of non-climbing palms, but not climbers (**Table 2**). Paleoclimatic changes (anomalies) since the Pliocene (PLIO_PREC_) and Miocene (MIO_PREC_) showed negative effects on species richness of both climbers (**Figures 3D,E**) and non-climbers (**Table 2**). This indicated that areas that were relatively wetter during the late Pliocene or late Miocene tend to have more palm species today than areas that were relatively drier in the past. In contrast to Pliocene and Miocene climate variables, precipitation and temperature anomalies since the LGM were not important to explain species richness of climbing palms, but the LGM_TEMP_ effect was important for non-climbing palms (**Table 2**).

**Table 2:** Multiple-predictor regression models to explain global species richness of climbing (*n* = 534) and non-climbing (*n* = 1911) palms.

		Palm species richness				
		Climbers			Non-climbers	
		Standard coefficient	*P*		Standard coefficient	*P*
Intercept		2.798	***		2.413	***
CANOPY		**0.403**	**		**0.227**	*
CANOPY^2^		NA			0.063	n.s.
PREC		**-**			0.271	n.s.
TEMP		**0.421**	***		**0.539**	***
PREC SEAS		**-0.356**	*		0.219	n.s.
PREC SEAS^2^		NA			0.044	n.s.
TEMP SEAS		**-**			**-0.507**	**
TEMP SEAS^2^		**-**			NA	
PREC DRY		**-**			NA	
PREC DRY^2^		NA			NA	
TEMP COLD		**-**			NA	
TEMP COLD^2^		**-**			NA	
LGM_PREC_		**-**			**-**	
LGM_PREC_^2^		**-**			NA	
LGM_TEMP_		**-**			0.220	n.s.
PLIO_PREC_		**-0.274**	*		**-**0.070	n.s.
PLIO_PREC_^2^		NA			**-0.201**	*
PLIO_TEMP_		**-**			**-**	
MIO_PREC_		**-**0.183	n.s.		**-**0.001	n.s.
MIO_PREC_^2^		NA			**-0.190**	*
MIO_TEMP_		**-**			**-**	
REGION						
Afrotropics		**-1.359**	***		**-1.098**	***
Australasia		**-1.786**	***		0.078	n.s.
Neotropics		**-1.773**	***		0.658	n.s.
Oceania		NA			**-**0.057	n.s.
Nearctic		NA			**-**0.854	n.s.
Palearctic		NA			**-0.855**	*
*AIC*		200			443	
*R^2^*		0.69			0.70	
Moran’s *I*		**-**0.13	n.s.		0.11	n.s.

Based on the AIC, a minimum adequate model was selected from the full set of predictor variables after removing those variables that showed high multicollinearity. Standardized coefficients are given for continuous predictor variables (scaled before the analysis to mean = 0 and SD = 1). The effect of the categorical variable REGION is relative to Indomalaya. Statistically significant effects of continuous and categorical variables are highlighted in bold. Sampling units are International Working Group on Taxonomic Databases (TDWG) level 3 units (*n* = 82 for climbers; *n* = 164 for non-climbers). ‘–’ indicates not selected variables, ‘NA’ not available variables or categorical levels. Residual spatial autocorrelation was tested using Moran’s *I* values based on the closest neighbor of each TDWG level 3 unit. Species richness and several continuous predictor variables (TEMP, PREC SEAS, TEMP SEAS, PREC DRY, LGM_TEMP_) were log_10_ transformed. Abbreviations of predictor variables: CANOPY, canopy height; PREC, annual precipitation; TEMP, annual mean temperature; PREC SEAS, precipitation seasonality; TEMP SEAS, temperature seasonality; PREC DRY, precipitation of driest quarter; TEMP COLD, Mean temperature of coldest quarter; LGM_PREC_, Last Glacial Maximum precipitation anomaly; LGM_TEMP_, Last Glacial Maximum temperature anomaly; PLIO_PREC_, Pliocene precipitation anomaly; PLIO_TEMP_, Pliocene temperature anomaly; MIO_PREC_, Miocene precipitation anomaly; MIO_TEMP_, Miocene temperature anomaly; REGION, biogeographic region (categorical variable, effects are relative to the Indomalayan region). Significance levels: ***P < 0.001; **P < 0.01; *P < 0.05; n.s., not significant. *R^2^*, explained variance.

The minimum adequate models included CANOPY as an important predictor variable for the species richness of both non-climbing and climbing palms, but the effect was stronger for climbers than for non-climbing palms (**Table 2**). The CANOPY effect on climbing palm species richness was positive (**Figure 3A**), supporting the hypothesis that richness increases with canopy height of forests. In addition to CANOPY and contemporary climate as well as paleo-climatic variables, biogeographic region (REGION) was also selected in the minimum adequate models (**Table 2**). Climbers showed a significantly higher species richness in Indomalaya relative to other tropical regions (**Figure 3F**) whereas non-climbers showed a significantly lower species richness in the Afrotropics relative to Indomalaya and the Neotropics (**Table 2**). These results were similar to the trends in raw species richness among realms (**Figure 2**), but they additionally accounted for major differences in contemporary climate, canopy height, and paleoclimate. This suggests that deep-time historical effects beyond those driven by Quaternary and late Neogene climate contribute substantially to the major differences in species richness of climbers and non-climbers among regions.

### DIVERSIFICATION ANALYSES

Comparing the ten ClaSSE diversification models revealed that model 7, the cladogenetic speciation and extinction model, was the best fitting model based on average AIC_c_ values from the 100 trees (**Table 3**). This was not only the case for the unconstrained analysis but also for the constrained analysis. This model suggested that the anagenetic state change rates between climbing and non-climbing palms are equal (q_1-0_= q_0-1_, **Figure 1**), while all other parameters are significantly different. Climbers had on average significantly higher speciation and extinction rates when compared to non-climbers (**Table 3**).

**Table 3:** Inferred rates of speciation and extinction for climbing and non-climbing palms using the Cladogenetic State Speciation and Extinction model (ClaSSE) for the best fitting model (model 7) out of ten (see **Figure 1**).

Model	df	logL	AIC_c_	λ_000_	λ_001_	λ_011_	λ_100_	λ_110_	λ_111_	μ_0_	μ_1_	q_01_ = q_10_
Model 7 (non-constrained)	9	-5582	11182	0,64	0,00004	0,00015	0,00021	0,00002	0,87	0,61	0,8	0,00002
SE				0,011	0,00001	0,00001	0,00004	0,00001	0,094	0,012	0,097	0
Model 7 (constrained)	9	-5768	11554	0,53	0,00002	0,0002	0,00022	0,000001	0,58	0,485	0,491	0,00001
SE				0,00504	0,000008	0,00001	0,00005	0,000001	0,022	0,005	0,025	0.000002

*In model 7 (non-constrained analysis), simulated crown nodes were allowed to vary between 0 and 100% of the whole length of the subtending branch of the genus. In model 7 (constrained analysis), the crown node of genera were restricted to be between 20 and 100% of the whole length of the subtending branch of the genus (see Materials and Methods). df, degrees of freedom; LogL, likelihood of model; AIC_c_, Akaike Information Criterion (AIC) correction for finite sample sizes.*

The BAMM analyses reached a stationary state well before 100,000 generations in all independent runs. The ESS values for the number of shifts and the log-likelihood were always above 200, indicating appropriate sampling of parameters from the posterior. Under the Poisson prior of 1.0, the zero rate shift model was rarely sampled from the posterior, strongly supporting the hypothesis of diversification rate heterogeneity across palms. Based on a Poisson prior of 0.1, the most probable number of rates shifts was 9 (PP = 0.135), closely followed by 8 (PP = 0.132), and 10 (PP = 0.131). This means that diversification rates have changed 9, 8, or 10 times across the history of palms.

**FIGURE 4:**
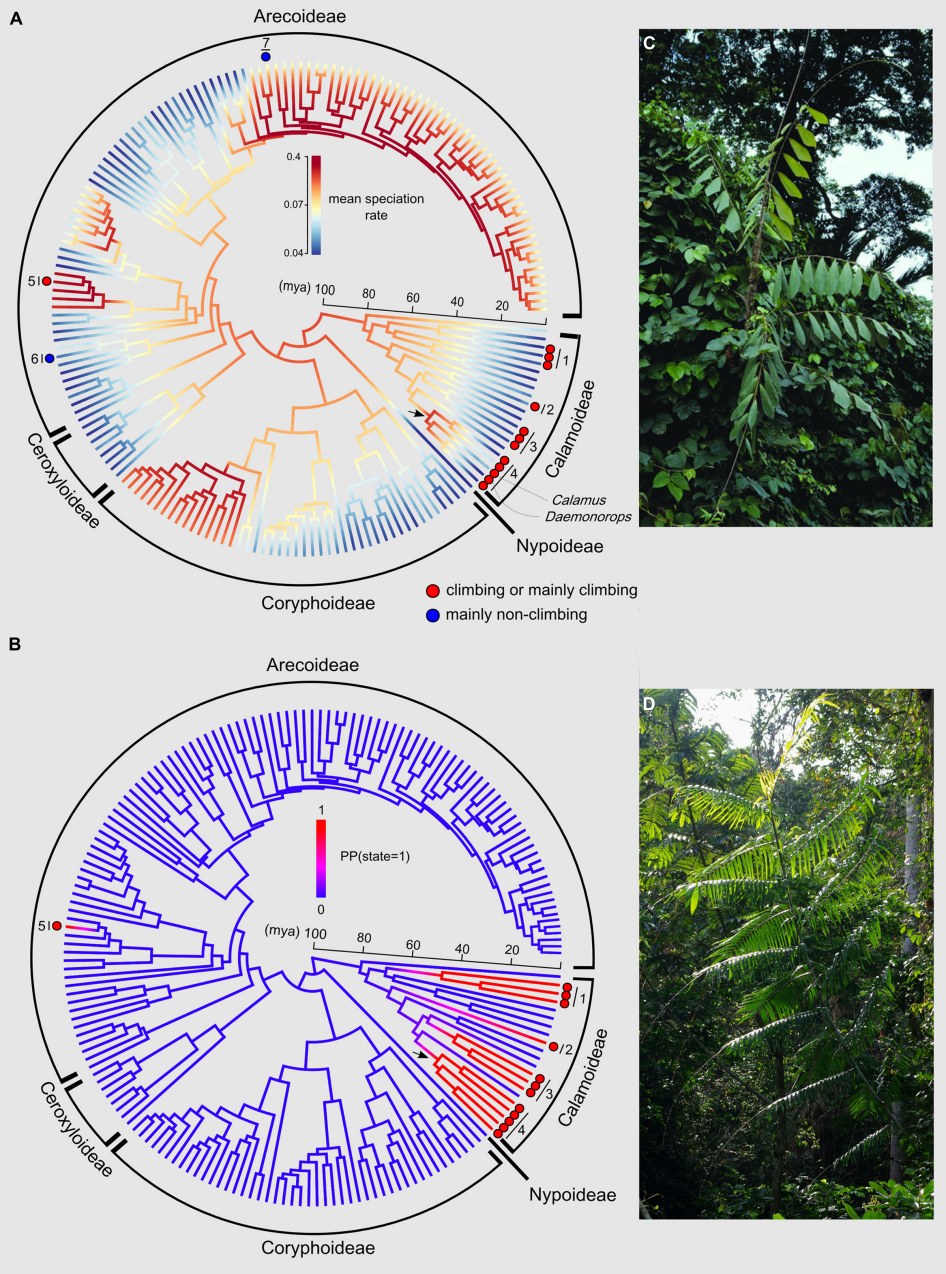
**Evolutionary history of the climbing habit in palms. (A)** Phylorate plot of the mean diversification rates sampled from the posterior (red = high diversification rates; blue = low diversification rates) resulting from the BAMM output using the dated generic-level chronogram (Couvreur et al., 2011a) in millions of years ago (mya). Each terminal branch represents a genus. The figure also shows the phylogenetic distribution of genera that include climbing species either containing entirely or mainly climbing species (red circles) or mainly non-climbing species (one or two species of climbers, blue circles). All other non highlighted genera are strictly non-climbers. The arrow indicates the crown node of subtribe Calaminae. Numbers refer to subtribes or genera (1: Ancistrophyllinae; 2: Korthalsiinae (*Korthalsia*); 3: Plectocomiinae; 4: Calaminae; 5: *Desmoncus*; 6: *Chamaedorea*; 7: *Dypsis*. **(B)** DensityMap plot of the posterior probabilities (PP) of state 1 (climbing, blue) vs. state 0 (non-climbing, red) along branches based on 10,000 generations using SIMMAP. The arrow indicates the crown node of subtribe Calaminae. The five state changes from non-climbing to climbing are represented by a change in color from blue to red. Photos illustrate typical climbing palms: **(C)** *Korthalsia zippelii* from Papua New Guinea (Photo: William J. Baker, www.palmweb.org), and **(D)** *Laccosperma robustum*, a frequent species throughout Central Africa (Photo: Thomas L. P. Couvreur, www.palms.myspecies.info).

The phylorate plot shows an increase in mean diversification rates at the crown node of subtribe Calaminae (depicted by an arrow in **Figure 4A**). Calaminae is the largest clade of climbing palms containing around 20% of all palms species (Dransfield et al., 2008). We can also note an increase in diversification rates for subtribe Bactridinae, which contains one genus of climbing palms (*Desmoncus*). Other climber dominated clades do not show such increases.

**FIGURE 5:**
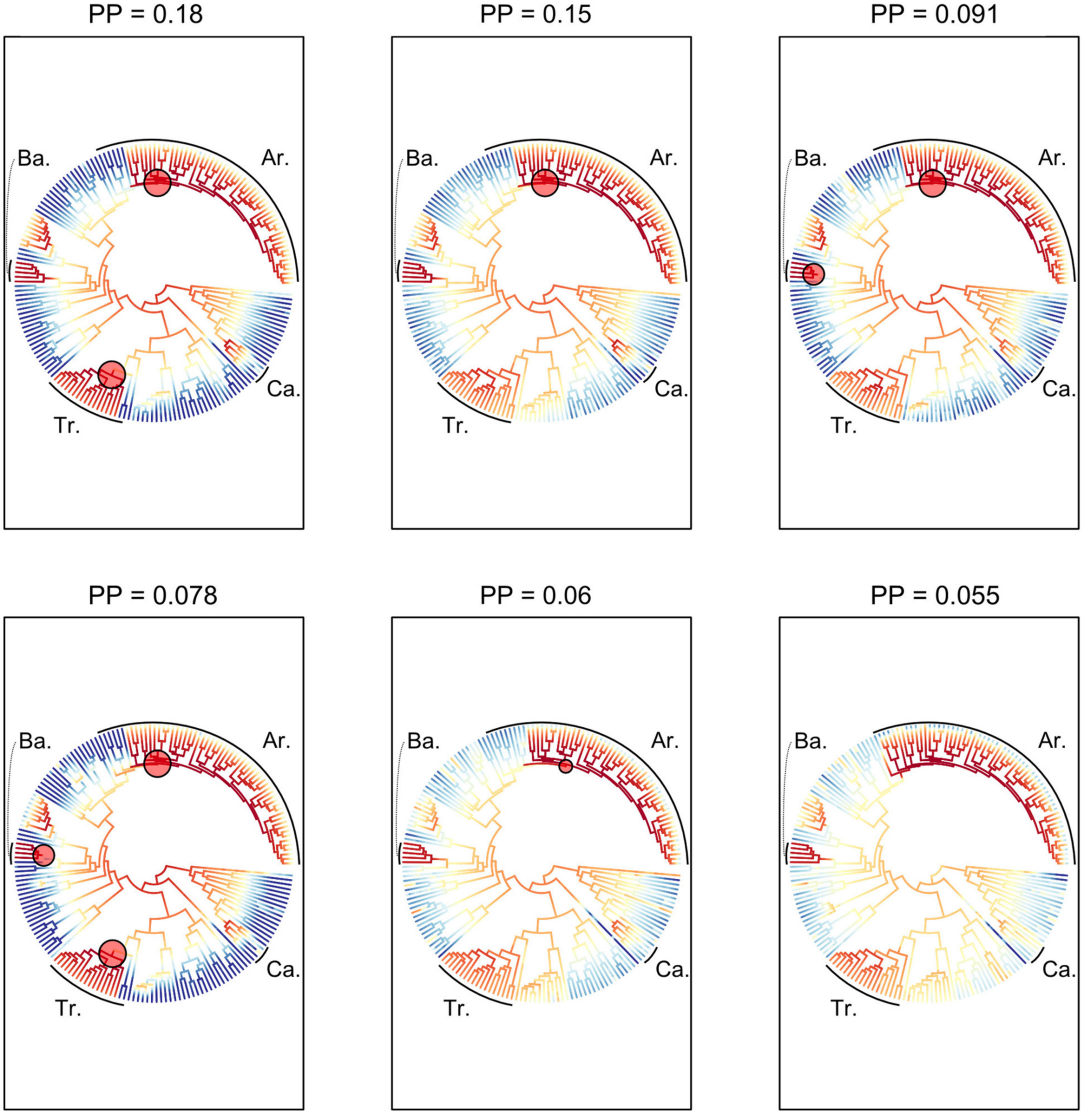
**Most probable combination of diversification rate shifts across palms.** The six most credible rate shift sets (CSS) with the highest posterior probability using a Bayes factor (BF) threshold of 50 based on the dated generic-level chronogram (Couvreur et al., 2011a). For each distinct shift configuration, the locations of diversification rate shifts are shown with filled circles (red = rate acceleration). Circle size is proportional to the marginal probability of that shift. Letters highlight clades that are associated with significant rates shifts (Ba, Bactridinae; Tr, Trachycarpeae; Ar, Areceae). The increase in diversification rates leading to the most species-rich clade of climbing palms, subtribe Calaminae (Ca), was not significant within the first six CSS.

**FIGURE 6:**
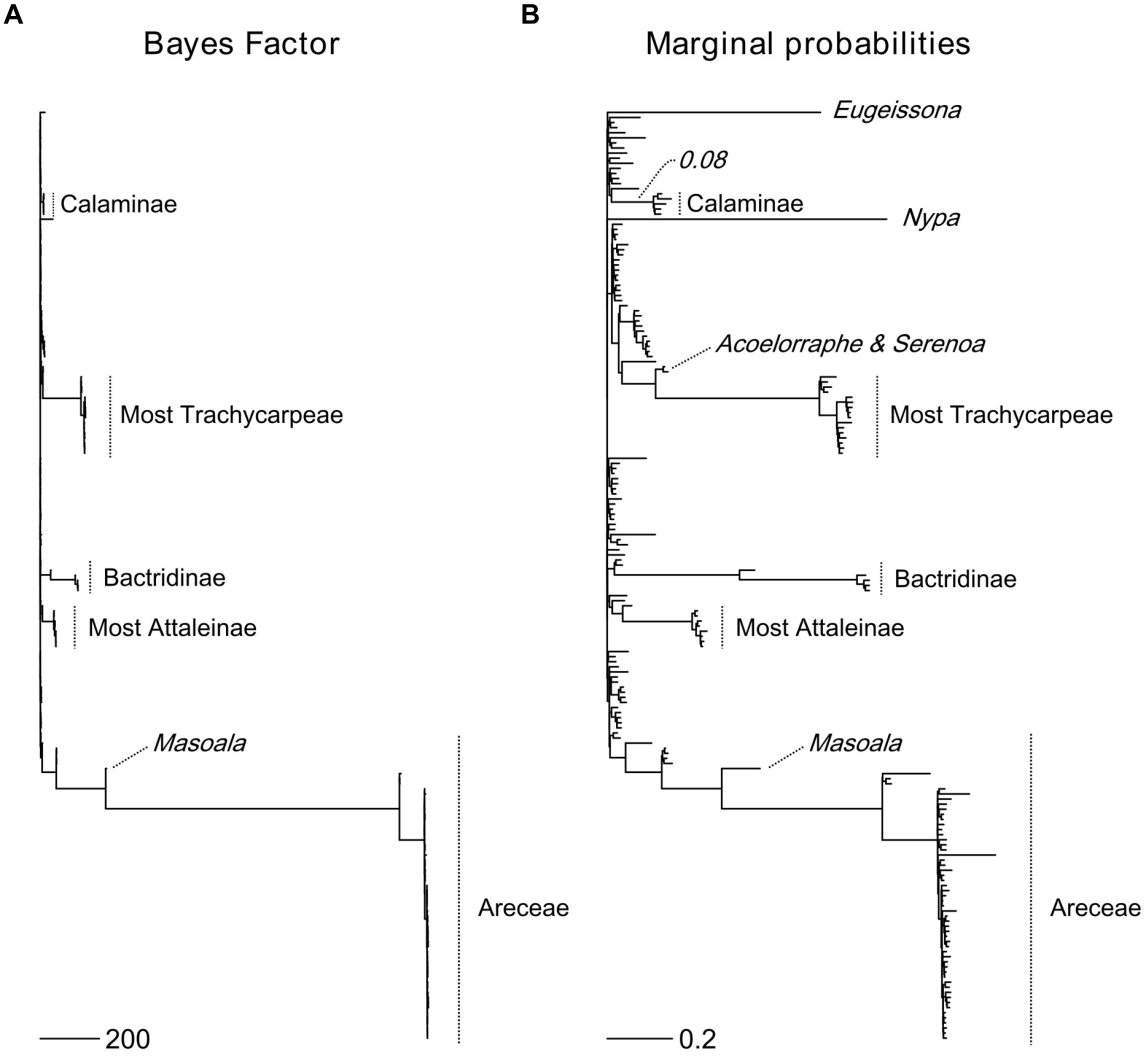
**Topological location of diversification rate shifts across palms.** The Bayes Factor (BF) **(A)** and marginal probabilities **(B)** of rate shifts are proportional to branch lengths. Major palm clades associated with rate shifts are highlighted. For subtribe Calaminae, the largest clade of climbing palms, a shift probability is visible along its root branch in both cases, but it is not significant when compared to other rate shifts in palms. Scale bars represent the value of the branch lengths for the BF **(A)** and the marginal probabilities **(B)**.

The six most probable CSS’s (with a cumulative PP of 0.614, **Figure 5**) show that significant rate increases (red circles) are mainly located on the branches leading to part of the tribe Areceae, most of tribe Trachycarpeae and most of subtribe Bactridinae. No significant rate shifts within theses sets were identified in relation to climbing dominated clades mainly found within Calamoideae (**Figure 5**). **Figure 6** represents the phylogenetic tree of palms where branch lengths were scaled to their BF (a) or marginal probabilities (b) of containing a rate shift. The shift in probability for subtribe Calaminae is visible in both cases but not significant when compared to other branches across palms.

### ANCESTRAL CHARACTER RECONSTRUCTION

Posterior mapping identified 4.9 average transformations from the non-climbing to the climbing state across the 100 trees, and 0.61 transformations in the opposite direction (reversals). These results do not include the three climbing species within the genera *Dypsis* and *Chamaedorea*, but in terms of timing of the origin of climbers in palms this omission has no effect on the results. The ancestral state of the crown nodes of the subtribes Calaminae, Plectocomiinae, and Ancistrophyllinae were strongly supported as climbing [PP(1) = 1.00; 0.99; 0.98, respectively]. **Figure 4B** shows that the climbing habit first evolved in palms along the branch leading to the crown node of subtribe Ancistrophyllinae during the early Eocene (55–47 mya). A second and third origin of the climbing habit was dated to the late Eocene and early Oligocene (44–33 mya) along the branches leading to the crown nodes of the subtribes Plectocomiinae and Calaminae. The timing of the two last origins could not be estimated, having evolved along the stem node of the South American genus *Desmoncus* and the Southeast Asian genus *Korthalsia* (**Figure 4B**).

## DISCUSSION

### ENVIRONMENTAL AND GEOGRAPHIC CORRELATES OF CLIMBING PALM SPECIES RICHNESS

Our results show that climbing palm species richness is associated with present-day climate (temperature, precipitation seasonality) and paleoclimatic changes since the late Neogene (Miocene and Pliocene; **Table 2**; **Figure 3**). An increase in species richness with higher temperatures was observed for both climbers and non-climbers which is in line with our hypothesis (prediction 1a) and consistent with findings on global palm diversity patterns (Kissling et al., 2012a). This relationship reflects the limited physiological and functional adaptations of palms, which reduce their survival in areas with cold climates (Tomlinson, 2006). However, the missing effect of temperature seasonality and the observed negative effect of precipitation seasonality on species richness of climbers are opposite to that of non-climbers (**Table 2**). The negative correlation with precipitation seasonality also contrasts with woody climbing plants, which increase (rather than decrease) in diversity with precipitation seasonality (Schnitzer, 2005). These trends could be explained by climbing palms being mostly found within Calamoideae, a subfamily that is constrained to warm and humid environments (Baker et al., 2000c; Couvreur et al., 2011a). The effect of precipitation changes (rather than absolute levels of contemporary precipitation) is also supported by the paleoclimatic effects in our regression models, which showed that Miocene and Pliocene precipitation anomalies had negative effects on climbing palm species richness. Hence, areas that were relatively wetter during the late Miocene or late Pliocene today have more climbing palm species than areas that were drier in the past. This suggests that multimillion-year non-equilibrium dynamics in diversity–climate relationships play a role in explaining present-day diversity of palms (Blach-Overgaard et al., 2013).

Our results further support the hypothesis that climbing palms are more diverse in tall-stature forests than in lower canopy ones (**Figure 3A**; hypothesis and prediction 1b). Forest canopy is not uniform across continents, being highest in Southeast Asia and Africa when compared to South America and Australia (Banin et al., 2012). Indeed, higher canopies and larger trees could arguably provide a three dimensional space that is more effectively exploited by certain kinds of climbing plants, particularly climbing palms. First, higher forests have a more patchy canopy because they have larger gaps in between tree trunks and fewer large trees reaching maximum heights (e.g., dipterocarp forests, see below, Richards, 1996). Second, larger trees can lead to larger tree-fall gaps, which has been shown to maintain a higher diversity of climber species in general (Putz, 1984; Schnitzer and Carson, 2001). However, in contrast to non-monocot lianas (Schnitzer, 2005) the majority of climbing palms might be less tolerant of strong disturbances. This is probably also reflected in the negative relationship of palm species richness with PREC SEAS (**Table 2**), which is opposite to dicotyledonous lianas (Schnitzer, 2005). Third, climbing palms have developed particularly long and light stiff “searcher-stems” compared with many other woody climbers, and they also produce long and sharp-toothed cirri and flagella. These climbing traits are particularly effective for spanning large gaps between large and tall canopy trees.

As indicated by Isnard and Rowe (2008), there is relatively little developmental and anatomical difference between climbing and non-climbing palms compared with clades of woody species in which climbers can develop highly derived and complex stem structures via anomalous secondary growth. Since the arguably more “simple” palm organization can develop effective “liana”-sized climbers it is intriguing that palms have not evolved the climbing habit more often and more widely, especially in the Neotropics. With just seven species, the Neotropics contain few representatives of the subfamily Calamoideae (Dransfield et al., 2008) and the observed diversity anomaly among regions could thus be related to phylogenetic constraints within palms (Bjorholm et al., 2006). Compared with the Calamoideae, few climbers have evolved within the Arecoideae (the largest palm subfamily in general and also within the Neotropics, Pintaud et al., 2008; **Figure 4**). This is reflected in genera such as *Chamaedorea* and *Dypsis* (Madagascar), which each contain one and two climbing species out of 110 and 140 species, respectively (Dransfield et al., 2008). The failure to diversify as climbers might be related to the fact that they are non-spiny, in contrast to the majority of other climbers in the Calamoideae. The only exception in Arecoideae is the Neotropical spiny genus *Desmoncus* (**Figure 4**), which has a currently reported diversity between 12 and 24 species (Isnard et al., 2005; Henderson, 2011). Other tropical plant families show a similar pattern of low climber diversity in the Neotropics compared to the Paleotropics. For example, Annonaceae, an important TRF plant family (Couvreur et al., 2011b), have a single climbing species in the Neotropics compared to ∼500 species in the Paleotropics. This might point toward potential phylogenetic constraints on certain plant families to successfully evolve and diversify as climbers in a given region. Some families might be particularly successful in their evolution toward a climbing form in one region, for example, the Neotropics (e.g., Bignoniaceae; Lohmann et al., 2013) whereas others might not (e.g., palms and Annonaceae).

### THE EVOLUTIONARY DIVERSIFICATION OF CLIMBING PALMS

Evidence suggests that the evolution of climbers promoted diversification within angiosperms (Gianoli, 2004). The diversification history of palms has not been homogenous across time (Baker and Couvreur, 2013b), and our results based on an improved diversification analysis approach (Rabosky, 2014) confirms this hypothesis. Indeed, a total of nine rate shifts across palms were detected with the highest posterior probability.

The analyses presented here suggest an important evolutionary role of climbers in explaining present-day palm diversity (hypothesis 2). Our ClaSSE analyses indicated that across palms, species with a climbing habit diversified on average 1.3 times faster when compared to species with a non-climbing habit (λ_111_ > λ_000_; see **Table 3**, prediction 2a). Elevated diversification rates for particular clades are highly dependent on young estimated crown node ages (Linder, 2008). In our case, young crown node ages of large climbing genera such as *Calamus* and *Daemonorops* will strongly pull toward higher speciation rates for climbers compared to non-climbers. An inverse result would occur if crown node ages were inferred to be very old for both these genera. Unfortunately, to date no valid estimations of crown nodes exist for genera within Calaminae. By constraining the crown node ages of all genera in our simulations to be between 20 and 80% of the stem node age (‘constrained analysis’), we avoided bias due to very old or very young crown node ages. Under these conditions, the climbing habit was also found to have significantly higher speciation rates, albeit slightly smaller in magnitude (**Table 3**).

Despite the overall higher diversification rate associated to climbers, the impact of the climbing trait on the diversification of specific clades is unclear (prediction 2a). Based on the analysis of the BAMM output, an increase in diversification rates (**Figure 4A**) is detected around the crown node of subtribe Calaminae (subfamily Calamoideae), the most species rich clade of climbing palms with around 500 Southeast Asian species (Dransfield et al., 2008). Surprisingly, however, this increase is not significant when compared to other diversification rate shifts found across the family (**Figures 5** and **6**). Nevertheless, a previous diversification study using the same phylogenetic tree and data but based on a ML stepwise AIC approach implemented in MEDUSA identified two significant rate increases at the stem nodes of *Calamus* and *Daemonorops*, both genera belonging to Calaminae (Baker and Couvreur, 2013b). In addition, *Calamus* was found to have significantly more species than expected under a constant birth–death rate model as well as have significantly higher diversification rates when compared to other genera under a high extinction rate assumption (Baker and Couvreur, 2013b). Even though these results should be interpreted with caution, taken together they support the idea that the evolution of the climbing habit in Calaminae positively impacted diversification rates in palms resulting in the speciation of a fifth of all palm species.

The increase in diversification rates in Bactridinae does not appear to be related to the climbing habit that evolved in the genus *Desmoncus*. Indeed, this rate increase, which was also detected with the MEDUSA analysis (rate shift 8 of Baker and Couvreur, 2013b that included *Desmoncus*, *Bactris*, and *Astrocaryum*), concerns most of this subtribe (excluding *Acrocomia*, **Figure 5**) and was suggested to be related to the evolution of epidermal spines, a common trait of all Bactridinae members which functions as a protection against herbivory in the Neotropics (Baker and Couvreur, 2013b).

The ancestral state reconstructions indicated that the climbing habit evolved a minimum of five independent times in palms: four times in Calamoideae and once in Arecoideae (**Figure 4B**). These results are consistent with the inferences for Calamoideae of Baker et al. (2000a). However, the climbing habit arose at least seven independent times because of the three climbing species within *Dypsis* and *Chamaedorea* that were not taken into account in the analysis presented here (both genera coded as non-climbing). At least in *Chamaedorea*, the single climbing species (*C. elatior*) is nested within the genus validating this assumption (Cuenca and Asmussen-Lange, 2007). In addition, these independent evolutions will be valid if our coding assumptions of *Calamus* and *Daemonorops* as ancestrally “climber” are also correct. Indeed, both these genera have a small proportion of non-climbing species (**Table 1**), and in this study we did not take them into account (both genera coded as climbers). The phylogenetic relations within both genera and for the subtribe Calaminae in general remain insufficiently understood and the exact placement of the non-climbing species is unresolved (Baker et al., 2000b).

Our results reveal an interesting pattern: the climbing habit appears to have had an impact on diversification rates in Calaminae, but not in other clades/genera where it evolved (Ancistrophyllinae, Plectocomiinae, *Korthalsia*, *Desmoncus*). For example, the climbing subtribes Ancistrophyllinae and Plectocomiinae (**Figure 4A**) have relatively old stem node ages associated with few extant species (Dransfield et al., 2008; Sunderland, 2012; Baker and Couvreur, 2013a; Faye et al., 2014). Why did the climbing habit have such an important effect on the diversification in a particular clade and not in other clades? One reason might be that different morphological adaptations are underlying the “same” trait (Donoghue, 2005), i.e., that convergent evolution (homoplasy) is constructed quite differently in different lineages and therefore has different impacts on diversification rates. For instance, in palms the climbing habit in different clades is associated with different combinations of morphological characters (Baker et al., 2000a; Isnard, 2006). Comparative studies of anatomical characters between Calaminae (*Calamus* and *Daemonorops*) and *Desmoncus* and subtribe Plectocomiinae (although based on the study of a few species) suggest that the evolution of a more flexible stem within subtribe Calaminae could explain the outstanding diversification of this group in terms of climbing mechanics (Rowe et al., 2004; Isnard, 2006). In addition, the evolution of a unique climbing organ (the flagellum, a modified inflorescence) has taken place within *Calamus* (Dransfield et al., 2008) and might have provided an additional advantage over other climbing structures in palms. Moreover, the presence of a “knee” a swelling at the junction of the leaf sheath and petiole, in most species in subtribe Calaminae and, to some extent, the African genus *Eremospatha* has been suggested as a potentially important trait for enhancing leaf strength at this important stress point (Isnard and Rowe, 2008). Overall, the repeated homoplasious occurrence of the climbing habit in the Calamoideae (‘clustered homoplasy’) could also indicate a more cryptic evolutionary innovation related to genetic or developmental precursors (Marazzi et al., 2012). In subfamily Calamoideae, this could be related to the tendency of organizing epidermal emergences as whorls, which manifest themselves, for example, both in the characteristic scales on the fruits as well as in organized grapnel spines on climbing organs. Finally, some life history traits might also act against increasing diversification rates. Indeed, Plectocomiinae and the genera *Korthalsia* (Korthalsiinae, **Figure 4C**) and *Laccosperma* (Ancistrophyllinae, **Figure 4D**) are hapaxanthic (individual stems die after a single flowering event), a condition which is generally not associated with high species richness across palms (Dransfield et al., 2008). Thus, several morphological novelties of subtribe Calaminae absent from other climbing genera (more flexible stems, the evolution of a flagellum in *Calamus*, the better mechanical role of the leaf sheath under stress, and the presence of a knee) possibly played decisive functional roles in explaining the diversification of this group when compared to other climbing palms.

Explaining geographic differences in diversification rates (e.g., numerous climbing species in Southeast Asia vs. few in the Neotropics) might be related to the fact that a particular trait only increases diversification rates under certain environmental conditions (de Queiroz, 2002). For instance, a highly dynamic geographic setting linked to a complex biogeographic history of Southeast Asia (Hall, 2009) might be important in explaining the extraordinary diversification of rattans in this region (Baker and Couvreur, 2012). The estimated increase in diversification rates around the crown node of subtribe Calaminae (34 Ma, **Figure 4A**) coincides with a period of important geological activity (Oligocene, early Miocene) induced by the collision of Sundaland and Australia (Hall, 2009). The Oligocene–Miocene boundary was also an important climatic transition going from a seasonally dry to an everwet aseasonal climate (Morley, 2007). In line with our results, numerous other studies have underlined the importance of the Miocene in the diversification of the Southeast Asian flora (Morley, 2007; Su and Saunders, 2009; Lohman et al., 2011; Nauheimer et al., 2012; Thomas et al., 2012; Bacon et al., 2013; Buerki et al., 2013; Richardson et al., 2014).

One important and characteristic plant family in Southeast Asia is Dipterocarpaceae (Appanah and Turnbull, 1998), with major peaks in diversity in Malaysia and mainland Southeast Asia. Its species are generally large emergent trees, which often dominate the upper canopy of the region’s forests, contributing to the much taller stature of the forests in this region relative to, e.g., the Neotropics (Richards, 1996). Interestingly, the first record of the family Dipterocarpaceae in Southeast Asia (Borneo) after its dispersal from India (Dutta et al., 2011) as well as the start of its estimated radiation in the region (Morley, 2000) correspond to the timing of increased diversification rates within the Calamoideae (Oligocene to early Miocene). We therefore hypothesize that the diversification of dipterocarps in combination with the evolution of several morpho-anatomical traits in Calaminae species could have triggered the radiation of climbing palms in this region. Ecological opportunity responses of one clade based on the success of another have been suggested in other cases such as ferns (Schneider et al., 2004) or ants (Moreau et al., 2006) during the radiation of angiosperms.

## CONCLUSION

Global diversity patterns of climbing palms show a diversity anomaly relative to other palms, with a strong peak of species richness in Southeast Asian rain forests and low species richness in other regions (e.g., the Neotropics). Present-day climate, forest canopy heights, and paleoclimatic changes in the Neogene and Quaternary can partly explain this pattern, but they do not provide a sufficient explanation for the extraordinary diversification of climbing palms in Southeast Asia. An increase in diversification rates in Calaminae, even though not significant based on our data, relative to other climbers and non-climbers might instead be the outcome of anatomical and morphological innovations, the complex biogeographic history of Southeast Asia, and/or ecological opportunity responses to the regional presence and diversification of tall canopy trees such as dipterocarps. We suggest that, in addition to climatic and paleoclimatic factors, such historical and evolutionary contingencies play an important role in explaining present-day biodiversity across TRFs. New datasets (e.g., global high-quality species distribution data at fine resolutions, well resolved species-level phylogenies, and additional morphological trait data) as well as novel analytical tools will likely increase our knowledge of palm diversification and our understanding of tropical rain forest evolution in the future.

## Conflict of Interest Statement

The authors declare that the research was conducted in the absence of any commercial or financial relationships that could be construed as a potential conflict of interest.

## Footnotes

1 http://pmip2.lsce.ipsl.fr/

2 https://stat.ethz.ch/pipermail/r-sig-phylo/2012-January/001826.html

3 http://bamm-project.org/postprocess.html
